# Collaborative research efforts benefit both Primarily Undergraduate Institution faculty and students and a biotechnology company in reproducibility project

**DOI:** 10.17912/micropub.biology.001604

**Published:** 2025-05-14

**Authors:** Kristen C Johnson, Lori Hensley, Christin Pruett, Lyndsay Avery, Roslyn Crowder, Laura Diaz-Martinez, Rebecca Giorno, Audrey Kim, Paul Kim, Adriana LaGier, Jamie Newman, Elizabeth Padilla-Crespo, Nik Tsotakos, Nathan Reyna

**Affiliations:** 1 University of New Hampshire Manchester, Manchester, NH USA; 2 Jacksonville State University, Jacksonville, Alabama, United States; 3 Ouachita Baptist University, Arkadelphia, Arkansas, United States; 4 Saint Michael's College, Colchester, Vermont, United States; 5 Stetson University, DeLand, Florida, United States; 6 Gonzaga University, Spokane, Washington, United States; 7 Louisiana Tech University, Ruston, Louisiana, United States; 8 Grambling State University, Grambling, Louisiana, United States; 9 Grand View University, Des Moines, Iowa, United States; 10 Universidad Interamericana de Puerto Rico, Recinto de Aguadilla; 11 Pennsylvania State University, State College, Pennsylvania, United States

## Abstract

Undergraduate students often have limited access to industry-focused research opportunities. To address this, faculty and students from 10 primarily undergraduate institutions collaborated with Sampling Human, a biotechnology company, to test a biocytometry workflow for single-cell analysis. The project engaged 15 students with varying levels of research experience and demonstrated that prior research expertise was not essential for successfully using the workflow. Participants followed standardized protocols and generated reproducible data comparable to that of PhD-level scientists. Despite some technical challenges, 91.7% of participants completed the study, showcasing the approachability and reliability of the workflow. This collaboration highlights the potential of industry partnerships to expand research opportunities, enhance academic visibility, and foster academic-corporate co-publications.

**Figure 1. Educational Experiences Based on Collaborative Efforts Between PUI Faculty, Students, and a Biotech Company:**
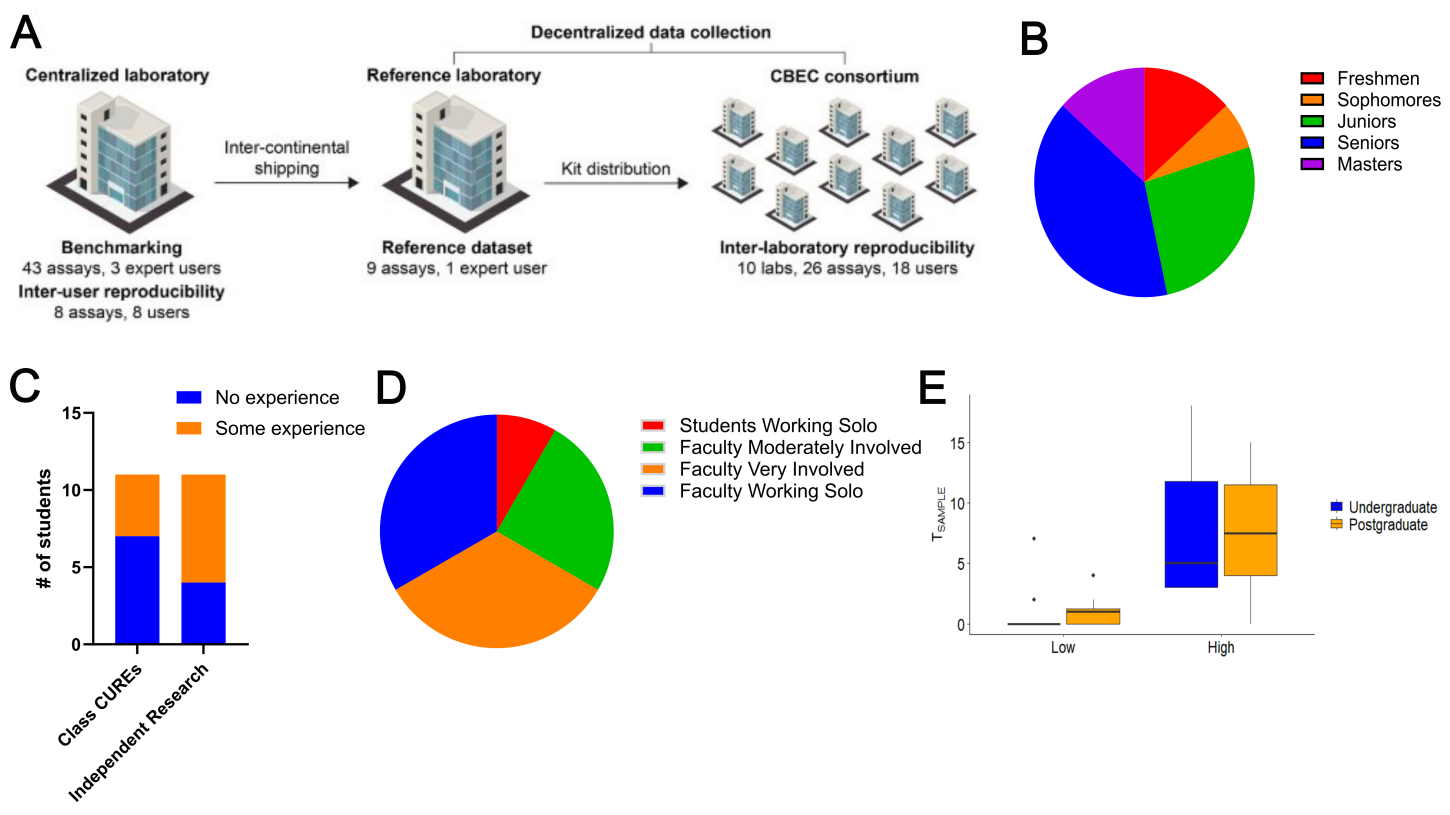
(A) Study design. The figure outlines the distribution of biocytometry kits and HUMO samples and the number of assays carried out by various laboratories. Distribution of 84 assays from the study dataset: 78 assays encompassing HUMO samples of every type complemented by 6 complementary assays from the inter-user reproducibility study encompassing high HUMO samples only. 18 CBEC participant users include 15 students as well as 3 faculty users. ( *Adapted from Figure 1C;* Fikar et al., 2024). (B) Make-up of CBEC student participants; (C) Prior research experience of the CBEC student participants; (D) Faculty interaction with CBEC students during reproducibility experiments; (E) Undergraduates and postgraduates detected differences between low and high numbers of targets (F=39.390, df=1, 48, P = 9.48 X 10 ^-8^ ) and there was no difference in their ability to detect targets (F=0.051, df=1,48, P = 0.822). We found no interaction between test type (low, high) and user group (undergraduate, postgraduate) (F=0.102, df=1,48, P = 0.751)

## Description

Some undergraduate students at primarily undergraduate institutions (PUIs) have access to traditional research opportunities in their university lab settings throughout their academic training or through internal or external summer research opportunities. Over time, different mechanisms have been established, many through course-based experiences (CREs) and larger inclusive Education Research Communities (iRECs), to enable more student access to genuine research experiences. However, not as many students and even fewer PUI faculty are exposed to collaborative work with industry. Only some students experience an industry internship at some point in their training due to a variety of reasons including accessibility and limited number of opportunities available. Bringing industry into undergraduate research settings addresses accessibility and enables PUI faculty and students to have meaningful interactions with an industry partner. In fact, it has been documented that on the national level academic-corporate collaboration and co-publishing enhances academic visibility with increases in the field-weighted citation impact (from 1.29 no collaboration to 2.5 with corporate collaboration) and citations per publication (from 13.8 no collaboration to 27.4 with corporate collaboration) ( *Collaboration Metrics - SciVal* , n.d.). Thus, industry collaboration can positively impact and expand upon the typical research experience for PUI faculty and students.

Here, we describe student participation in a reproducibility project of a new product for Sampling Human (Berkeley, CA). Sampling Human is a TechBio company whose goal is to develop novel technologies for single-cell analysis inspired by groundbreaking research in synthetic biology. Cell Biology Education Consortium (CBEC) is a National Science Foundation Research Coordination Network in Undergraduate Biology Education whose Principal Investigators (PIs) connected with the Sampling Human co-founder at the iGEM Grand Jamboree 2022 (Paris, France) which was the foundation of the collaboration described.

Utilizing the Sampling Human biocytometry workflow, students (and faculty) at Primarily Undergraduate Institutions (PUIs) followed written and video protocols to process samples provided by the Sampling Human Reproducibility Project. The study aimed to ensure the reproducibility and accuracy of their product designed for single-cell research, through a large-scale, multi-center inter-laboratory experiment, thereby showcasing the potential in overcoming user, hardware and location variability to generate consistent, reliable and reproducible results. Using Diagnostics on Target (DOT) bioparticles capable of targeting and reporting the presence of cells based on their cell surface markers, the concentrations of EpCAM positive HaCaT cells among a background of EpCAM negative HL-60 cells in three different human mockup (HUMO) samples were measured by all reproducibility study participants (Fig1A).

Sampling Human chose to collaborate with the CBEC to capitalize on our network and ready access to faculty and students from primarily undergraduate institutions enthusiastic to participate in the beta testing. Twelve faculty from ten PUIs that are part of the CBEC collaborated with Sampling Human to receive kits to beta test the biocytometry workflow with their students. In total, 15 students at various levels took part in the experimentation (Fig 1B). Throughout the process, students and faculty met with the Sampling Human team to review the protocol, equipment to be used, and the data acquisition. The prior research experience of the students varied from those having no independent research experience and/or no experience in a classroom undergraduate research experience (CURE) setting to those having one or both experiences (Fig 1C) showcasing that there was not a requirement for prior experience in a laboratory/research environment when carrying out the experiments in the biocytometry assay.

Participating faculty were surveyed related to their involvement in the Sampling Human reproducibility project. Although faculty reported some challenges including 7 of 12 with some technical challenges (lack of appropriate equipment to complete protocol) and 4 of 12 with experience-based challenges (unfamiliarity with techniques/reagents/equipment being used in protocol), 91.7% of participating faculty were able to complete the experimentation and submit data to Sampling Human for final analysis.

While in one case undergraduate students worked completely on their own for the experimentation, there was a variety of faculty involvement with students during the process of carrying out the testing (Fig 1D). This range reflects some of the timing and logistics of the experiments and may also reflect some of the technical challenges with carrying out the experiments as one faculty noted that despite much troubleshooting and working with multiple company representatives their school’s plate reader was not compatible with the first version of the kit precluding student involvement. Nevertheless, faculty reported positive interactions with their students and successful efforts when working in teams. For example, one faculty stated: “[I] Worked with them together the first time and checked in with them frequently during repeated attempts. They worked together when I wasn't there.” Another faculty commented: “I ran through the protocol alongside another student who then assisted another group performing the study.” Finally, a third faculty reflected: “Project worked well for us. I did not do much work getting the students involved. My senior was amazing and did not need much attention. Eventually she [my sophomore] became the leader of the project. [She] came in on her own to teach a freshman how to run the lab. This was a success, but the plate reader broke and we were unable to collect more data.”

After completion of the biocytometry assay, CBEC faculty and students submitted their plate reader data to Sampling Human for final analysis and assessment of reproducibility of their assay in the field. Sampling Human provided data to compare the statistical differences between data produced by PhD level scientists and that from students and faculty at PUIs. Both undergraduates and postgraduates detected differences between low and high numbers of targets (F=39.390, df=1, 48, P = 9.48 X 10 ^-8^ ) and there was no difference in their ability to detect targets (F=0.051, df=1,48, P = 0.822). We found no interaction between test type (low, high) and user group (undergraduate, postgraduate) (F=0.102, df=1,48, P = 0.751) (Fig 1E). In addition, CBEC students participating in the reproducibility project testing performed equally as well with the research protocol as PhD level scientists performing benchmarking (centralized Sampling Human laboratory) and reference experimentation (reference laboratory) (Fig 1A, Fikar et al., 2024). This emphasizes the technical skills of the students at PUIs and the quality, suitability, and reproducibility of the Sampling Human biocytometry product.

Having set the stage for a successful collaboration and meaningful data generation, PUI faculty from the CBEC continue to work with Sampling Human on other biocytometry assays that are conducive to the PUI setting. It is evident that these kits are not only approachable for undergraduate students and their faculty, but they generate reproducible data using equipment that is available at many undergraduate institutions. This collaboration enabled Sampling Human to complete their reproducibility project and publish a manuscript collaboratively with the CBEC faculty and student participants. We are excited about sharing these resources and industry collaboration with the greater PUI community through our CBEC QUBEShub community page ( https://qubeshub.org/community/groups/cellbioed ). This type of collaboration continues to expand the typical research experience for PUI students and faculty, enabling industry collaboration, participation in research protocols, and co-publication.

## Methods

All biocytometry reagents were provided by Sampling Human and the experiment was carried out according to the published protocols (Fikar et al., 2024).

**Statistical Analysis:** A two-way ANOVA was performed within R ( *R: The R Project for Statistical Computing* , n.d.) to evaluate differences in test detectability (log T _sample_ ) between low and high numbers of cells and between undergraduate students and postgraduates. A boxplot was created to compare T _sample_ values between groups and between tests using the package ggplot2 (Wickham, 2016) in R. The total number of target cells in the sample analyzed, T _SAMPLE_ , is the sum of T _WELL_ values across its 8 respective wells.
